# An Improved Deep Residual Convolutional Neural Network for Plant Leaf Disease Detection

**DOI:** 10.1155/2022/5102290

**Published:** 2022-09-14

**Authors:** Arun Pandian J., Kanchanadevi K., N. R. Rajalakshmi, G.Arulkumaran

**Affiliations:** ^1^Department of Computer Science and Engineering, Vel Tech Rangarajan Dr. Sagunthala R and D Institute of Science and Technology, Chennai, India; ^2^School of Computing and Information Technology, Reva University, Bengaluru, India

## Abstract

In this research, we proposed a novel deep residual convolutional neural network with 197 layers (ResNet197) for the detection of various plant leaf diseases. Six blocks of layers were used to develop ResNet197. ResNet197 was trained and tested using a combined plant leaf disease image dataset. Scaling, cropping, flipping, padding, rotation, affine transformation, saturation, and hue transformation techniques were used to create the augmentation data of the plant leaf disease image dataset. The dataset consisted of 103 diseased and healthy image classes of 22 plants and 154,500 images of healthy and diseased plant leaves. The evolutionary search technique was used to optimise the layers and hyperparameter values of ResNet197. ResNet197 was trained on the combined plant leaf disease image dataset using a graphics processing unit (GPU) environment for 1000 epochs. It produced a 99.58 percentage average classification accuracy on the test dataset. The experimental results were superior to existing ResNet architectures and recent transfer learning techniques.

## 1. Introduction

Agriculture is an important sector for many countries and provides raw resources for many businesses [1]. Diseases, insects, and nutrient deficiencies are the most common threats to the growth of crops. Disease diagnosis and treatment, pest management, and fertiliser application are performing an important role in decreasing yield loss [2]. The traditional process for disease detection is not feasible for all crop fields and farmers. Finding suitable human experts for disease diagnosis and treatment requires more time and money. An artificial intelligence approach is required for the automatic detection of plant diseases to overcome difficulties in the traditional approach [3].

Deep learning is a type of artificial intelligence technique that extends from artificial neural networks [4]. The deep learning technique imitates how humans make intelligent decisions through acquiring knowledge [5]. It is increasingly being used in various industrial applications for decision support to increase productivity, reduce errors, and reduce costs. Deep learning techniques perform better than traditional artificial intelligence techniques in terms of decision accuracy and reliability [6]. Deep convolutional neural networks (DCNN) are a class of supervised deep learning techniques. The DCNNs are most successful in image classification and object detection tasks [7]. A large volume of data is required to train the DCNN models for use in various domains [8]. The data augmentation technique was introduced to increase the amount of training data without data collection for better training performance of DCNN models [9]. Training the DCNN model needs huge computation and storage.The graphics processing units (GPUs) are commonly used to train models more efficiently [10].

The major contributions of this research are as follows:

- The leaf diseases of twenty-two different plants were diagnosed using novel deep residual convolutional neural networks.
- A novel deep residual convolutional neural network with 197 layers (ResNet197) was designed and developed for leaf disease detection.
- In addition, the evolutionary searching technique was used as a tuning technique to discover the suitable number of layers and hyperparameter values for the proposed ResNet197 model.
- ResNet197 was trained on the plant leaf disease dataset up to 1000 epochs in a GPU environment.
- The classification performance of trained Resnet197 was calculated on the test dataset using standard performance metrics.
- This research also proposed a model that could be used by farmers for diagnosing various plant diseases from a camera-captured image without any prior knowledge of plant diseases.
- Performance comparison of the proposed model and recent transfer learning techniques showed that it is superior to other transfer learning methods in leaf disease detection tasks.

The research article is organized as follows: In Section 2, we provided a brief study about plant leaf disease detection using various machine learning and deep learning approaches. In Section 3, the data preparation, the ResNet197 architecture, and the corresponding training process were presented. In Section 4, we experimentally compared the performance of ResNet197 with recent deep transfer learning techniques and discussed the outcomes. Finally, we concluded the research by summarizing the outcomes and future directions in Section 5.

## 2. Literature Survey

The recent developments in artificial intelligence techniques support efficient identification of numerous diseases and pest attacks in precision farming. This survey discusses the modern artificial intelligence approaches to plant leaf disease detection. In [11], the authors compared the performance of standard machine learning and deep transfer learning techniques in plant leaf disease detection. They identified that the performance of the deep learning techniques was better than that of machine learning techniques in leaf disease detections. The VGG-16 net produced a classification accuracy of 89.5% on plant leaf disease detection, which is higher than that of other machine learning and deep learning techniques.

The authors in [12] proposed a DCNN with nineteen convolutional layers for the classification of two major apple leaf diseases. The classification accuracy of the model on test data for apple disease detection was 99.2%. The model produced a better performance than support vector machine (SVM), k-nearest neighbour (K-NN), random forest (RF), and logistic regression (LR) techniques. On the other hand, the authors in [13] used a capsule network with a bidirectional long short-term memory model for the classification of apple leaf diseases. The classification performance of their model was better than that of the standard machine learning techniques. Also, the ensemble subspace discriminant analysis classifier with a mask region-based convolutional neural network was used to detect the infected regions of apple crop leaves by the authors in [14]. They achieved a classification accuracy of 96.6% on the tomato leaf disease dataset using their model.

The authors in [15] used a dense convolutional neural network (DenseNet) and multilayer perceptron for detecting bacterial leaf blight, brown spot, and leaf smut diseases in rice crops. The maximum classification accuracy of the rice disease detection model was 97.68%. In [16], the authors proposed a rice crop disease detection model using an attention-based neural network and MobileNet. The rice crop disease detection model has classified the diseases with an accuracy of 94.65% on the test data. The authors in [17] developed a VGG16Net-based rice and wheat leaf disease detection model. The rice disease and wheat disease classification accuracy of the model was 97.22% and 98.75%, respectively. They compared the performance of the model in rice and wheat disease detection with that of other transfer learning techniques.

Likewise, the authors in [18] designed a simple DCNN to diagnose tomato crop diseases, and they achieved a 98.49% of classification accuracy on testing data. In [19], the authors developed a tomato leaf disease detection model using the DenseNet121 transfer learning technique. They used the conditional generative adversarial network (C-GAN) for creating augmented data for balancing training datasets. The DenseNet121 model achieved an accuracy of 97.11% on tomato disease classification. In [20], the authors proposed a custom convolutional neural network for plant disease classification. The custom network achieved a classification accuracy of 94.5% on the test dataset. The authors in [21] developed an EfficientNet pretrained model for detecting peach plant diseases with an accuracy of 96.6% on the test data. The improved MobileNet model was proposed for cassava disease detection by the authors in [22]. Also, they achieved better performance than other machine learning and transfer learning techniques in cassava leaf disease detection using MobileNet.

Similarly, the authors in [23] proposed a cucumber leaf disease severity classification model using U-Net architecture and achieved a testing accuracy of 92.85% on the cucumber leaf disease dataset. In [24], the authors proposed a pumpkin powdery mildew disease identification technique using principal component analysis (PCA) and SVM. The model detected the pumpkin powdery mildew disease on the pumpkin leaf with an accuracy of 97.3%, and the authors in [25] developed a cotton lesion detection model using the Resnet50 transfer learning technique. The model produced a classification accuracy of 89.2%, which is better than that of GoogleNet and standard machine learning techniques. Moreover, the authors in [26] developed a super-resolution generative adversarial network (SR-GAN) as an augmentation technique for balancing the data numbers in classes of the dataset.

Also, they identified that the augmented dataset increases the classification accuracy of deep learning models. A custom DCNN model with nine layers was proposed to identify the diseases of thirteen different species by the authors in [27]. The model classified 96% of the images accurately in the test dataset. Recently, the authors in [28] proposed a custom DCNN model for the detection of plant leaf diseases on the standard dataset and field-collected images. The custom DCNN model achieved an average testing accuracy of 99.84% on the test dataset. The authors in [29] proposed a DenseNet architecture for the diagnosis of the twenty-seven different classes of diseases from six crops. The validation and testing accuracy of the classification model was 99.58% and 99.19%, respectively. The authors in [30] proposed a custom network for detecting pearl millet diseases. They achieved an accuracy of 98.78%, which is higher than that of the transfer learning techniques. The authors in [31] studied various plant leaf disease detection techniques using a deep convolutional neural network. Also, they discussed several datasets, which are available for plant leaf disease detection model development.

The literature survey recognized that residual and dense convolutional neural networks performed better than other transfer learning techniques in plant disease detection [32]. The residual and dense network created deeper connections between the layers than simple convolutional neural networks. The residual and dense networks avoided the vanishing-gradient problem and minimized the number of training parameters. The performance of the residual and dense network in existing plant leaf disease detection applications provided the motivation to propose a residual convolutional neural network for plant leaf disease detection. Most of the state-of-the-art transfer learning techniques were trained on the ImageNet dataset. The transfer learning techniques may cause negative transfer and overfitting problems while using the architecture and weights of the pretrained models for new applications.

In addition, the literature survey shows the significance of data augmentation and hyperparameter tuning for the classification algorithms. A novel residual convolutional neural network was proposed in this research with improved performance than existing residual networks and other transfer learning techniques for detecting plant diseases. The subsequent section discussed the architecture and training process of the proposed plant disease detection model.

## 3. Materials and Methods

The proposed plant leaf disease detection model implementation steps are classified into two stages. Implementation of the proposed ResNet197 model started with the data preparation. The data preparation phase concentrates on data collection, augmentation, and data preprocessing. The model training phase includes ResNet197 design, fine-tuning, and training processes. The following subsections describe each of the implementation phases in detail.

### 3.1. Data Preparation

Implementation of a deep learning algorithm starts with the data preparation phase. It includes data collection, data augmentation, and preprocessing stages. The proposed dataset was collected from various standard leaf disease detection datasets [27, 32]. There are 103 classes of healthy and diseased images in the proposed dataset.  Table 1 illustrates the list of diseased and healthy plant leaf classes in the proposed dataset.

**Figure 1:**
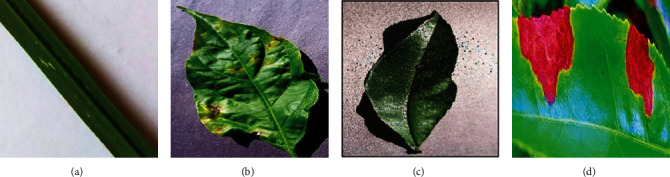
Sample augmented images from the plant leaf disease dataset.

Some classes in the original dataset have fewer samples. On the other hand, some classes have more images. For example, the tea leaf blight disease class has only 214 images, but the tomato yellow leaf curl virus disease classes have 3209 samples. The number of samples should be equal in each class to increase the performance of the classification algorithms. Data augmentation techniques were used in this research to increase the number of samples without collecting new data. The scaling, cropping, flipping, padding, rotation, affine transformation, saturation, and hue transformation techniques were used to produce augmented images on the dataset. The data augmentation process equalized the number of images in each class to become 1500.  Figure 1 shows the sample augmented images on the plant leaf disease dataset using data augmentation techniques.

After the augmentation step, the dataset was split for the training, validation, and testing process. The images in the dataset were shuffled and randomly selected for training, validation, and testing. The number of images in the training, validation, and the testing dataset is illustrated in Table 2.

The training process of the proposed ResNet197 model was discussed in subsequent sections. The training process includes model design, fine-tuning, and model training steps.

### 3.2. Model Training

**Figure 2:**
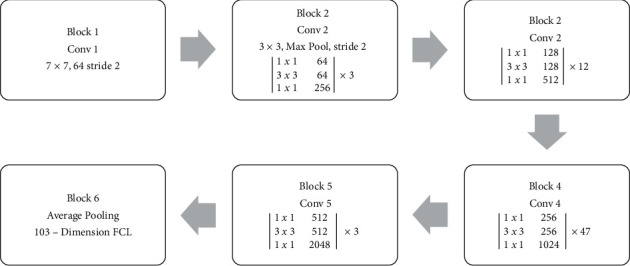
Layered architecture of the proposed ResNet197 model.

This section discussed the construction and training process of the proposed ResNet197 model for leaf disease detection. Six blocks of layers were used in the proposed model. Also, the proposed model was called a deep residual convolutional neural network with 197 layers (ResNet197). The proposed ResNet197 model includes 197 layers in total. The layered architecture of the proposed ResNet197 model is shown in Figure 2.

The input image size of the proposed ResNet197 model was 224 × 224 × 3 pixels. The first block consisted of one convolutional (Conv) layer. The first convolutional (Conv) layer produced 112 × 112 sized outputs using a 7 × 7 Conv function with a stride of 2. The convoluted data were forwarded to the second block. The second block consisted of one max-pooling layer and three Conv layers. The three Conv layers were used three times in sequence. The output of block 1 was forwarded to the max-pooling layer, which uses a 3 × 3 max-pooling function with a stride of 2. The output of the pooling layer was sent as an input to three Conv layers. The second layer block produced an output sized 56 × 56. The output of the second block was forwarded to the third block. The third layer block consisted of three Conv layers sized 1 × 1, 3 × 3, and 1 × 1 filter size. The Conv layers were used 12 times in a sequence. The third block produced an output sized 28 × 28. After the third block layer, the data were forwarded to the fourth layer block. Three Conv layers were available in the fourth block. The three Conv layers were used 47 times in a sequence. The fourth Conv layer produced the output data with a size of 14 × 14. The fifth layer block was introduced after the fourth block. Three Conv layers were used in the fifth block three times in a sequence. The fifth block produced the 7 × 7 sized output. The output of the fifth block was forwarded to the sixth and final block of the model. The sixth block consisted of an average pooling layer and one fully connected (dense) layer with 103 neurons. The softmax activation function was used in this layer for classifying the input leaf images.

The suitable batch size, loss function, optimizer function, and learning rate of the proposed ResNet197 model were identified using the evolutionary search technique.  Table 3 displays the optimised hyperparameter value of the proposed ResNet197 model.

**Figure 3:**
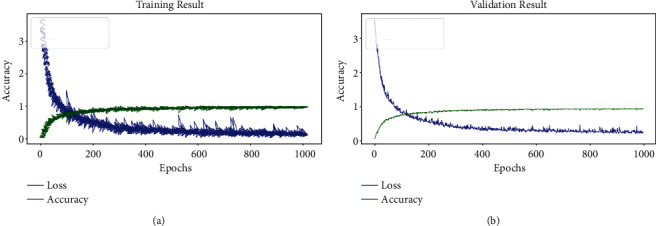
(a) Training and (b) validation results of ResNet197.

The proposed ResNet197 model was trained on the plant leaf disease dataset using the optimised hyperparameters up to 1000 training epochs. The training progress and validation progress of the proposed ResNet197 model are shown in Figure 3.

There was no significant change in the validation performance of ResNet197 after reaching 1000 epochs. So, the training process of the model was stopped with 1000 epochs in the GPU environment. The proposed ResNet197 model was deployed after the successful completion of the training process. The testing process of the proposed ResNet197 model was discussed in the upcoming section.

## 4. Results and Discussions

This section discussed the performance of the proposed ResNet197 model in plant leaf disease detection. Also, it compares the ResNet197 model with other versions of ResNet models and state-of-the-art transfer learning techniques using standard performance metrics. VGG-19 Net, ResNet-152, InceptionV3 Net, Mobile Net, and DenseNet201 are the state-of-the-art transfer learning techniques that are used for the performance comparison.

**Figure 4:**
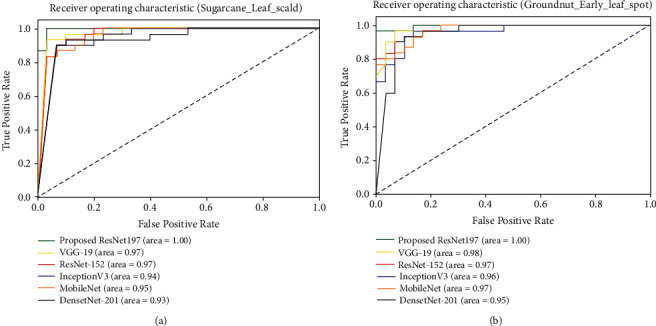
Sample AUC curves of ResNet197.

The area under the curve-receiver operating characteristics (AUC-ROC) curve is the most popular metric for estimating the performance of classification techniques. The ROC of classification techniques for a specific class is calculated using the true positive rate (TPR) and false positive rate (FPR) values of the class on the test data. The TPR represents the number of correctly classified positive samples in the test data [27]. Similarly, the FPR represents the number of incorrect positive predictions among negative samples in the test data. The TPR and FPR values are used to plot the ROC curve and calculate the AUC value of the classification model for a specific class. The *x*-axis and *y*-axis of the graph represent the scale of TPR and FPR, respectively. The AUC-ROC curves of proposed and existing models on two randomly selected classes are shown in Figure 4. The AUC values of ResNet197 on the sample classes were higher than those of other standard transfer learning techniques. The AUC value of the proposed ResNet197 model on the sample classes is between 0.98 and 1.0; it shows the performance excellence of ResNet197 on plant leaf disease classification.

**Figure 5:**
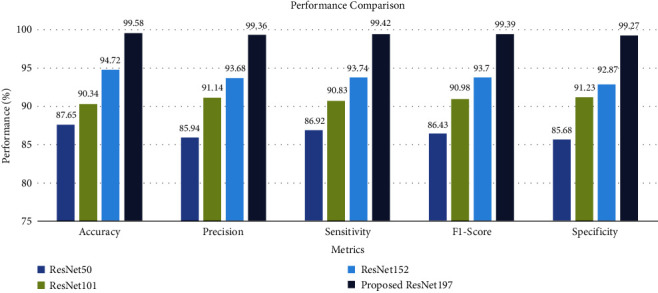
Performance comparison of ResNet architectures.

Classification accuracy, precision, recall, and F1-score are the standard measures to assess the overall performance of the classification techniques [27]. The performance of ResNet197 and most recent transfer learning techniques was compared using the abovementioned metrics. The performance comparison of the proposed ResNet197 model and transfer learning techniques is illustrated in Figure 5.

Also, Table 4 illustrates the performance comparison of the proposed ResNet197 model and other ResNet models.

**Figure 6:**
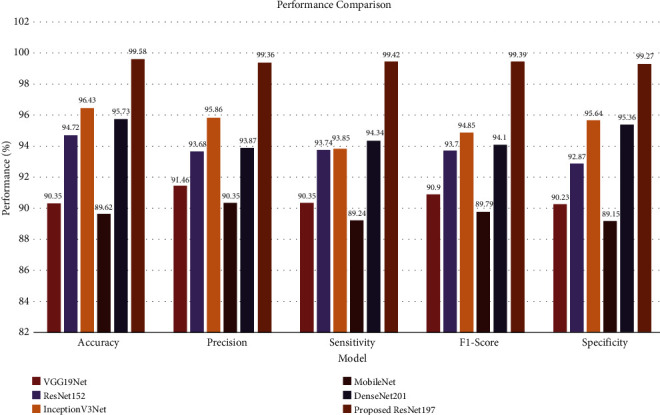
Performance comparison of ResNet197 and transfer learning techniques.

In addition, the classification performance of the proposed ResNet197 model was compared with that of existing state-of-the-art transfer learning techniques. The proposed model achieved an average classification accuracy of 99.58% on the test data. The performance comparison of the proposed ResNet197 model and transfer learning techniques using standard performance metrics is illustrated in Figure 6.

Also, Table 5 shows the performance score of the proposed and existing models on the plant leaf disease dataset. The comparison result shows that the proposed model achieved better classification accuracy, precision, sensitivity, F1-score, and specificity than existing transfer learning techniques.

The inceptionV3 network showed better performance among the transfer learning techniques in plant leaf disease detection. The average classification accuracy of the proposed ResNet197 model on the test dataset was 99.58%, which is 3.15% higher than that of the inceptionV3 network. The average classification accuracy, average precision, average recall, and average F1-score of the proposed ResNet197 model were superior to those of the other transfer learning techniques. The AUC values and performance metric outcomes of the proposed ResNet197 model showed that the performance and reliability of the proposed ResNet197 model were superior to those of advanced transfer learning techniques in plant leaf disease detection.

## 5. Conclusions and Future Works

Automatic plant disease detection is a crucial process in precision agriculture. This research study proposed a novel deep residual convolutional neural network with 197 layers (ResNet197) for the detection of common leaf diseases in 22 different plants. Some standard datasets and a few recent image augmentation techniques were used to prepare the proposed dataset for the ResNet197 training. Scaling, cropping, flipping, padding, rotation, affine transformation, saturation, and hue transformation techniques were used to produce the augmented images. The proposed dataset consisted of 133,900 images of 103 diseased and healthy classes. The evolutionary searching technique was used to identify suitable values for the hyperparameters of the proposed ResNet197 model in plant leaf disease detection. The training process of ResNet197 and existing transfer learning models was performed on GPU-enabled workstations up to 1000 training epochs. The classification accuracy, precision, sensitivity, F1-score and specificity of the proposed ResNet197 model were 99.58%, 99.36%, 99.42%, 99.39%, and 99.27%, respectively. The performance results of the proposed ResNet197 model were superior to those of the transfer learning techniques such as VGG19Net, ResNet152, InceptionV3Net, MobileNet, and DenseNet201. Also, AUC curves demonstrated the performance and reliability of ResNet197 in plant leaf disease detection. This research concludes that the deep residual convolutional neural networks with the optimised number of layer blocks perform better than traditional deep learning techniques. This research study also identified that the performance of the classification algorithms can be improved by data augmentation and hyperparameter optimization techniques. The limitation of ResNet197 is its computational density. It requires significantly more FLOPS than similar models such as VGG19Net and MobileNet. The development of a novel deep convolutional neural network using residually connected networks for the diagnosis of a number of plant diseases is a future direction of the research study.

## Figures and Tables

**Table 1:** List of classes in the proposed dataset.

ID	Class name
1	Aloe_Vera_Healthy
2	Aloe_Vera_Leaf_Rot
3	Aloe_Vera_Leaf_Rust
4	Apple_Black_Rot
5	Apple_Healthy
6	Apple_Leaf_Rust
7	Apple_Leaf_Scab
8	Banana_Bacterial_Wilt
9	Banana_Black_Sigatoka
10	Banana_Healthy
11	Banana_Mosaic
12	Carrot_ Alternaria Leaf Blight
13	Carrot_ Cercospora Leaf Blight
14	Carrot_ Sclerotinia Rot
15	Carrot_ Healthy
16	Cherry_Healthy
17	Cherry_Leaf_Rust
18	Cherry_leaf_Spot
19	Cherry_Powdery_Mildew
20	Citrus_Black_Spot
21	Citrus_Canker
22	Citrus_Greening
23	Citrus_Healthy
24	Citrus_Melanose
25	Coffee_Cercospora_Leaf_Spot
26	Coffee_Healthy
27	Coffee_Leaf_Rust
28	Coffee_Red_Spider_Mite
29	Corn_Common_Rust
30	Corn_Healthy
31	Corn_Leaf_Spot
32	Corn_Northern_Leaf_Blight
33	Corn_Southern_Leaf_Blight
34	Eggplant_Cercospora_Leaf_Spot
35	Eggplant_Healthy
36	Eggplant_Powdery_Mildew
37	Eggplant_Verticillium_Wilt
38	Grape_Black_Measles
39	Grape_Black_Rot
40	Grape_Healthy
41	Grape_Leaf_Blight
42	Groundnut_Early_Leaf_Spot
43	Groundnut_Healthy
44	Groundnut_Late_Leaf_Spot
45	Groundnut_Leaf_Rust
46	Groundnut_Web_Blotch
47	Guava_Algal_Leaf_Spot
48	Guava_Healthy
49	Guava_Leaf_Rust
50	Guava_Pseudocercospora_Leaf_Spot
51	Paddy_Bacterial_Blight
52	Paddy_Brown_Spot
53	Paddy_Cercospora_Leaf_Spot
54	Paddy_Healthy
55	Paddy_Hispa
56	Paddy_Leaf_Blast
57	Paddy_Leaf_Streak
58	Peach_Bacterial_Spot
59	Peach_Healthy
60	Peach_Leaf_Curl
61	Peach_Leaf_Rust
62	Pepper_Cercospora_Leaf_Spot
63	Pepper_Fusarium_Wilt
64	Pepper_Gray_Leaf_Spot
65	Pepper_Healthy
66	Potato_Early_Blight
67	Potato_Healthy
68	Potato_Late_Blight
69	Potato_Leaf_Roll
70	Potato_Potato_Virus_Y
71	Strawberry_Angular_Leaf_Spot
72	Strawberry_Healthy
73	Strawberry_Leaf_Scorch
74	Strawberry_Leaf_Scorch
75	Sugarcane_Eye Spot
76	Sugarcane_Red_Rot
77	Sugarcane_Pineapple_Disease
78	Sugarcane_Leaf_Scald
79	Sugarcane_Mosaic_Virus
80	Sugarcane_Healthy
81	Tea_Healthy
82	Tea_Leaf_Blight
83	Tea_Red_Leaf_Spot
84	Tea_Red_Scab
85	Tomato_Bacterial_Spot
86	Tomato_Early_Blight
87	Tomato_Healthy
88	Tomato_Late_Blight
89	Tomato_Leaf_Mold
90	Tomato_Leaf_Spot
91	Tomato_Mosaic_Virus
92	Tomato_Spider_Mite
93	Tomato_Target_Spot
94	Tomato_Yellow_Leaf_Curl_Virus
95	Turmeric_Bacterial_Wilt
96	Turmeric_Healthy
97	Turmeric_Leaf_Blotch
98	Turmeric_Leaf_Spot
99	Wheat_Bacterial_Leaf_Streak
100	Wheat_Healthy
101	Wheat_Leaf_Rust
102	Wheat_Powdery_Mildew
103	Wheat_Tan_Spot

**Table 2:** Size of training, validation, and the test dataset.

Dataset name	Number of images	Number of images in each class
Training set	133,900	1,300
Validation set	10,300	100
Testing set	10,300	100

**Table 3:** Optimized hyperparameter values of the ResNet197 model.

Hyperparameter	Optimized value
Batch sizes	64
Loss	Categorical cross entropy
Optimizer	Adam
Learning rate	0.001

**Table 4:** Performance comparison of ResNet models.

Model	Accuracy	Precision	Sensitivity	F1-score	Specificity
ResNet50	87.65	85.94	86.92	86.43	85.68
ResNet101	90.34	91.14	90.83	90.98	91.23
ResNet152	94.72	93.68	93.74	93.7	92.87
**Proposed ResNet197**	**99.58**	**99.36**	**99.42**	**99.39**	**99.27**

**Table 5:** Performance comparison of ResNet197 and transfer learning techniques.

Model	Accuracy	Precision	Sensitivity	F1-score	Specificity
VGG19Net	90.35	91.46	90.35	90.9	90.23
ResNet152	94.72	93.68	93.74	93.71	92.87
InceptionV3Net	96.43	95.86	93.85	94.85	95.64
MobileNet	89.62	90.35	89.24	89.79	89.15
DenseNet201	95.73	93.87	94.34	94.1	95.36
Proposed ResNet197	**99.58**	**99.36**	**99.42**	**99.39**	**99.27**

## Data Availability

The plant leaf disease data used to support the findings of this study are available from the corresponding author upon request.
